# Outcomes of Surgical Rib Plating: A Case Series

**DOI:** 10.7759/cureus.55446

**Published:** 2024-03-03

**Authors:** Ellie G Wallace, Jeremy Miller, Danielle Azani, Andrew McCague

**Affiliations:** 1 General Surgery, Western University of Health Sciences, Lebanon, USA; 2 Surgery, Desert Regional Medical Center, Palm Springs, USA; 3 General Surgery, Desert Regional Medical Center, Palm Springs, USA; 4 Trauma and Acute Care Surgery, Desert Regional Medical Center, Palm Springs, USA

**Keywords:** rib fixation, outpatient rib fracture, emergent general surgery, rib surgical fixation, trauma

## Abstract

Rib fractures are a common result of blunt thoracic trauma. Complications of rib fractures include pneumothorax, hemothorax, respiratory failure, and death. The conservative management of rib fractures has been the mainstay of care with surgical rib fixation as a secondary management only performed in complicated flail segments. The purpose of this retrospective study is to describe the outcomes of six patients who underwent surgical rib fixation following a traumatic injury at a Level 1 trauma center. All care for these cases was performed at Desert Regional Medical Center in Palm Springs, CA. On average, patients stayed 12.3 total days in the hospital and 4.6 in the intensive care unit. Out of the six patients, only one required prolonged respiratory support eventually resulting in respiratory failure and death. This retrospective study on surgical rib fixation highlights the importance of early surgical intervention and the need for more general and trauma surgeons to be familiar with the procedure itself.

## Introduction

Trauma is the leading cause of death in people under 40 years old, and one in four people dies because of traumatic injuries. The most common causes of traumatic injury are motor vehicle accidents, assault, and falls [1]. Common traumatic injuries include axial bone fractures, blunt head trauma, and blunt thoracic trauma. Thoracic trauma has a high mortality rate and is associated with a multitude of complications including pneumothorax, hemothorax, pulmonary contusions, blunt cardiac injury, and rib fractures [2].

Rib fractures have a high incidence of chest trauma and are typically managed in a conservative manner. Flail chest segments include chest wall fractures that result in the loss of stability around the chest cavity. Flail segments can be identified by the presence of paradoxical rib motion upon inspection of the chest wall as the lungs are unable to properly expand. Flail segments can be anterior, lateral, and posterior and can involve the sternum, costochondral junction, rib heads, and rib bodies [3]. The number of rib fractures typically correlates with both morbidity and mortality and higher rates of adverse outcomes including pneumonia, pneumothorax, and increased length of stay [4]. The first-line treatment of rib fractures has been symptom management including pain control, positive pressure ventilation, and pulmonary hygiene [2], with surgical rib fixation as a second-line management or reserved for complex chest traumas and severe flail segments [4].

Surgical rib fixation is the introduction of titanium plates that conform to the patients' ribs to stabilize them and prevent nonunion [2]. It is indicated in patients with chest wall instability and flailed chests, ventilated patients who fail to extubate, and patients undergoing a thoracostomy [4]. Absolute contraindications to this procedure are hemodynamic instability, atypical rib fractures, severe traumatic brain injury, and myocardial infarction [2]. Surgical rib fixation aims to reduce pain, improve ventilation mechanics, and allow for adequate expansion of the lungs [4].

The aim of this retrospective study is to present six cases, all of whom underwent surgical rib fixation following blunt thoracic trauma at a Level 1 trauma center. Here, we will outline the patients' mechanism of injury, which ribs were plated, and their outcomes including length of stay, days on the ventilator, post-op complications, and disposition.

## Materials and methods

We conducted a retrospective cohort study at Desert Regional Medical Center in Palm Springs, CA. Approval was obtained from Metro West Medical Center Institutional Review Board (approval number: 2023-101) prior to the initiation of this study. A chart review was completed to identify adult patients who had undergone blunt thoracic trauma resulting in multiple rib fractures and were treated with surgical rib fixation by a trauma surgeon at our Level 1 trauma center. Exclusion criteria were children, pregnant women, and prisoners. The data gathered for this study is routinely collected data at Desert Regional Medical Center over the course of a hospital stay, including demographics, history of present illness, past medical history, diagnostic imaging, length of hospital and intensive care unit stay, duration of ventilator requirement, diagnostic imaging, postoperative complications, and disposition. Data was collected onto a Microsoft Excel sheet and was subsequently used to calculate the Injury Severity Score and referenced to synthesize each case presentation.

## Results

Here, we present six cases in the past two years (four males, two females) ranging from 44 to 79 years old who presented with blunt thoracic trauma with multiple rib fractures that subsequently underwent surgical rib fixation.

Patient 1 is a 58-year-old male with no known past medical history who presented to the hospital after a motor vehicle crash in which he was the restrained driver who crashed into a median. Upon admission, the patient was found to be intoxicated with alcohol complaining of chest and back pain. Further diagnostic imaging revealed bilateral displaced rib fractures resulting in a flail chest, subarachnoid hemorrhage, pneumomediastinum, and a left pneumothorax. This patient was admitted for 12 total days, two of which in the intensive care unit. He underwent left chest tube placement, left anterolateral minithoracotomy, open reduction and internal fixation of the right radius and left pubic rami, and fixation of ribs 4-8 on the left with the KLS Martin fixation system. There were no postoperative complications, and the patient required no days on the ventilator. This patient was discharged to an acute rehabilitation unit without readmission within 30 days of his discharge.

Patient 2 is a 45-year-old male with no known past medical history who presented to the hospital following a high-speed dirt bike accident. The patient fell off his bike causing him to strike the right side of his chest. In the field, needle thoracostomies to the superior, anterior, and lateral chest were performed as the patient was noted to have decreased breath sounds over the right lung fields with shortness of breath. Upon arrival at the emergency department, the patient was hemodynamically stable. Diagnostic imaging showed right pneumothorax, pneumomediastinum, right flank contusion, shoulder hematoma, right clavicle, and rib 3-8 displaced fractures without a flail segment. He underwent a right anterolateral latissimus-sparing thoracotomy, evacuation of hemothorax, right upper lobe wedge resection, right lower lobe resection, closure of a right middle lobe laceration, mechanical pleurodesis, two right chest tube thoracostomies, and fixation of the right fifth and sixth ribs with the DePuy Synthes Matrix fixation system and self-drilling screws. There were no postoperative complications, and the patient required no days on the ventilator. This patient was discharged home after eight total days in the hospital, four of which were in the intensive care unit.

Patient 3 is a 44-year-old male with no known past medical history who presented to the hospital after a motorcycle accident. The patient was the helmeted driver of a motorcycle driving approximately 90 miles per hour when he was ejected. He suffered a 4-5-minute loss of consciousness and was transferred by air to the emergency department where he complained of left clavicle pain and left chest wall pain. Following further diagnostic imaging, he was found to have blunt head trauma, left hemopneumothorax, pulmonary contusions, and left clavicle, scapula, and rib 1-8 displaced fractures. This patient subsequently underwent a left anterolateral thoracotomy, left chest tube placement, and surgical fixation of the left sixth and seventh ribs with the DePuy Synthes Matrix fixation system and self-drilling screws. This patient required no days on the ventilator and sustained no postoperative complications. He spent 10 days in the hospital, two of which were in the intensive care unit, and was subsequently discharged home.

Patient 4 is a 58-year-old man with no known past medical history who presented to the hospital following a fall from 20 feet. The patient was found down next to a dumpster with a ladder next to it, and per EMS, he was complaining of head pain, right chest pain, and difficulty breathing. This patient sustained many injuries including right pneumothorax, mediastinal hematoma, right costochondral, sternochondral junction fracture, T1-T3 spinous process fractures, blunt head trauma, scalp laceration, and displaced fracture of right ribs 2-6 with a flail segment. He underwent median sternotomy, right thoracoscopic chest exploration, evacuation of hemothorax, costochondral fixation, and fixation of the right ribs 3-5 with the DePuy Synthes Matrix fixation system and self-drilling screws. This patient required three days on the ventilator and four days in the intensive care unit. He was discharged home after 12 total days in the hospital without any complications.

Patient 5 is an 85-year-old female with known arthritis, diabetes, and Parkinson's disease who was admitted to the hospital following a ground-level fall. Upon arrival at the emergency department, she complained of right shoulder pain and bilateral chest wall pain. Diagnostic imaging revealed a right proximal humerus fracture, left lung contusion, left hemothorax, and left rib 4-12 displaced fractures with a flail segment. This patient subsequently underwent left chest tube thoracostomy, left anterolateral thoracotomy, open reduction and internal fixation of the right humerus, and fixation of ribs 7-10 on the left with the DePuy Synthes Matrix fixation system and self-drilling screws. She spent no days on the ventilator and four days in the intensive care unit. After 11 days total in the hospital, this patient was discharged to a skilled nursing facility without any complications.

Patient 6 is a 79-year-old female with known atrial fibrillation and chronic back pain who presented to the hospital following a motor vehicle accident. The patient was the restrained front seat passenger in a vehicle traveling approximately 45 miles per hour. This patient suffered multiple injuries including blunt cardiac injury, blunt chest trauma resulting in left rib 4 and 6 displaced fractures without a flail segment and a sternal fracture, third metacarpal fracture, T7 vertebrae compression fracture, acute blood loss anemia, and atrial fibrillation with rapid ventricular response. She underwent multiple procedures including bilateral chest tube placement, left anterolateral thoracotomy, open fixation of the sternal fracture, fixation of the sixth rib with the DePuy Synthes Matrix fixation system and self-drilling screws, multiple intubations, bronchoscopy, and tracheostomy. Postoperatively, this patient was extubated and suffered respiratory failure requiring reintubation. She spent 21 total days in the hospital, 14 of which were in the intensive care unit, ultimately resulting in the withdrawal of care and death.

Table 1 shows the patients' demographics, mechanism of injury, and injuries.

**Table 1:** Patient demographics, mechanism of injury, and injuries M: male; F: female; BL: bilateral; R: right; L: left

Patient	Age	Sex	Past medical history	Mechanism of injury	Ribs plated	Flail segment	Other injuries
1	58	M	None	Motor vehicle accident	BL 4, 5, 6, 7, 8	Yes	Subarachnoid hemorrhage, pneumomediastinum, left pneumothorax
2	45	M	None	Dirt bike accident	R 5, 6	No	Right pneumothorax, pneumomediastinum, R clavicle fracture, R flank contusion, R shoulder hematoma
3	44	M	Alcohol use	Motorcycle accident	L 6, 7	Yes	Blunt head trauma, hemothorax, pneumothorax, L pulmonary contusion, L clavicle and scapula fracture
4	58	M	None	Fall	R 3, 5	Yes	R pneumothorax, pneumomediastinum, R costochondral and sternochondral junction fracture, T1-T3 spinous process fractures, L1 vertebrae fracture, blunt chest trauma
5	85	F	Parkinson's disease, arthritis, type II diabetes mellitus	Fall	L 7, 8, 9, 10	Yes	R proximal humerus fracture, L lung contusion, L hemothorax
6	79	F	Atrial fibrillation, chronic back pain	Motor vehicle accident	Sternum and 6	No	Blunt cardiac injury, blunt chest trauma, L patella fracture, R third metacarpal fracture, T7 compression fraction

Table 2 depicts the patients' outcomes status post-rib fixation.

**Table 2:** Patient outcomes status post-rib fixation LOS: length of stay; ICU: intensive care unit; ARU: acute care unit

Patient	LOS	ICU days	Ventilator days	Trach	Complications	Disposition
1	12	2	0	No	None	ARU
2	8	4	0	No	None	Home
3	10	2	0	No	None	Home
4	12	4	3	No	None	Home
5	11	4	0	No	None	Skilled nursing facility
6	21	12	12	Yes	Reintubated, readmitted to the ICU, respiratory failure, death	Expired

## Discussion

Blunt thoracic trauma occurs in 10-15% of all polytraumas, and 25% of those result in death [4]. Of the complications surrounding blunt thoracic trauma, rib fractures are the most common. Rib fractures in combination with other traumatic injuries are responsible for increased respiratory illness and overall mortality [4]. Although there is no literature that supports improved morbidity and mortality with rib fixation status post-thoracic trauma, there has been a correlation with reduced pain scale, length of stay, ventilator requirements, and other metrics of patient outcomes [5]. Here, we present six cases from a Level 1 trauma center, all of whom underwent blunt thoracic trauma and surgical rib fixation.

Of the six patients, on average, patients spent 12.3 days total in the hospital and 4.6 of those days in the intensive care unit. According to multiple large-scale retrospective studies, the average hospital length of stay for patients who have undergone blunt thoracic trauma is nine to 10 days [6]. Among our patient sets, case #6 is the most obvious outlier with 21 total days spent in the hospital. This may be attributed to this patient's complicated hospital course which included reintubation and the extended goals of care discussions with the patient's spouse who was also recovering from polytraumatic injury.

According to existing meta-analyses, there is a significant decrease in ventilator days in thoracic trauma patients who underwent surgical rib fixation [5]. Within our six cases, four patients spent no days on the ventilator, and only one of the patients required a tracheostomy. Patient 6 required 12 days total on the ventilator due to the complication of reintubation and eventual tracheostomy. This could be attributed to the patient's complicated thoracic injuries with a sternal fracture as well as multiple rib fractures, or it may be caused by a delay in surgical rib fixation. Of the surgeons at this trauma center, only two of the providers perform surgical rib fixations, and in this case, neither was available to perform the surgery until six to seven days after the initial insult. This highlights the importance for general and trauma surgeons to be aware of the benefits of early intervention in multiple rib fractures and become comfortable with the procedure in training.

Of the literature describing the effect of surgical rib fixation on morbidity and mortality, there is no clear picture of whether there is a statistically significant benefit [7]. This is likely caused by the limitations of these studies. Because of the nature of trauma, isolated multi-rib fractures are rare, and it is difficult to isolate the sole cause of death in patients who suffer polytrauma. Of the six cases we present in this paper, only one expired in the setting of withdrawal of care.

Of the six cases presented in this paper, only one had outlying complications and eventual death status post-surgical rib fixation. Of the patients who survived, their average hospital length of stay was slightly longer than the average reported in other studies, but they largely had great respiratory outcomes with none of them requiring long-term invasive interventions. Patient 6, the patient who expired, sheds light on the importance of more practitioners to be familiar with surgical rib fixation and the importance of early intervention as it may lead to better overall outcomes for the patient.

Limitations of this study include incomplete data collection and small sample size. The inability to follow these patients long-term in the clinic limits our ability to accurately measure the effect of rib fixation on morbidity and mortality. The sample size and nature of this study make it nearly impossible to generalize outcomes of rib fixation in blunt thoracic trauma. Therefore, larger studies must be conducted for the advancement of this technique in the field.

## Conclusions

Surgical rib fixation is an effective management of rib fractures in the setting of blunt thoracic trauma. Patients who undergo this intervention soon after the initial insult have better survivability and fewer complications related to rib fractures. They would also benefit from more providers becoming familiar with and willing to perform surgical rib fixation. Rib fractures and blunt thoracic trauma continue to result in high rates of morbidity and mortality; therefore, clinicians should have multimodal treatment options.
